# The effects of a stressed inshore urban reef on coral recruitment in Suva Harbour, Fiji

**DOI:** 10.1002/ece3.4641

**Published:** 2018-11-20

**Authors:** Ronal Lal, Stuart Kininmonth, Antoine D. R. N’Yeurt, Ralph H. Riley, Ciro Rico

**Affiliations:** ^1^ School of Marine Studies The University of the South Pacific Suva Fiji; ^2^ Centre for Ecology and Evolutionary Synthesis University of Oslo Oslo Norway; ^3^ Pacific Centre for Environment and Sustainable Development The University of the South Pacific Suva Fiji; ^4^ Western Washington University Bellingham Washington; ^5^ Instituto de Ciencias Marinas de Andalucía (ICMAN), Consejo Superior de Investigaciones Científicas Campus Univ. Río San Pedro 11510 Pedro Puerto Real (Cádiz) Spain

**Keywords:** Coral diversity, Coral recruitment, Inshore, Sedimentation

## Abstract

A relic inshore reef ecosystem adjacent to the Fijian capital of Suva and another remote inshore reef were monitored monthly from July 2014 to July 2015 for coral recruitment, sedimentation rates, coral cover, temperature, and light intensity. Despite a major sewage spill in Suva Harbour in December 2014, the municipal inshore site exposed to constant anthropogenic activity, recorded no significant differences in coral spat abundance (except for the family Poritidae) on artificial substrata compared to the remote inshore site. Total yearly spat abundance was 106 on municipal reef and 132 on remote reef, while average daily sediment trap collection rates (g cm^2^/day) were significantly higher in the municipal site for the entire duration of monitoring. Total annual particulate organic matter content in sediment was also significantly higher in the municipal site (107.51 g cm^2^), compared to the remote site (43.37 g cm^2^). Mean light intensity was significantly lower for the municipal site (69.81 lum/ft^2^) compared to the remote site (239.26 lum/ft^2^), with Photosynthetically Active Radiation also lower for the former (800–1,066.66 µmol m^−2^ s^−1^) compared to the latter (3,266.66–3,600 µmol m^−2^ s^−1^). The lack of significant differences in coral spat recruitment rates suggests that settling larvae may be unable to distinguish between sub‐optimal and optimal sites probably as a consequence of interference with coral settlement cues arising from anthropogenic development.

## 1 INTRODUCTION

The reproductive capacity of a reef ecosystem is vital to its sustainability (Bauman et al., 2015). The viability of its community structure is largely reliant upon the coral reef communities being either self‐seeding, where the coral larvae can be retained on a reef system through water flow patterns (Black, Moran, & Hammond, 1991), or dependent upon the transportation of larvae from adjacent reefs (Fisk & Harriott, 1990). The recruitment of coral juveniles is considered a major determinant of community structure in marine ecosystems and is recognized as a fundamentally important influence affecting the distribution and abundance of reef organisms (Babcock & Mundy, 1996; Harriott, 1992). The recruitment or re‐establishment of coral functional groups characteristic to a particular locality is also considered vital to the resilience, maintenance, and recovery of coral reefs (Bauman et al., 2015; Bellwood, Hughes, Folke, & Nystrom, 2004; Hughes, Graham, Jackson, Mumby, & Steneck, 2010). Coral recruitment rate is affected by a multitude of factors, some of which include depth, orientation of the substratum, competition with other organisms, grazing, availability of suitable habitat, and larval dispersal capabilities (Harriott, 1992). Specific coral larval settlement cues involving reef acoustics, chemical‐algal signals, reef surface parameters, and light are vital in influencing the orientation of coral planulae toward healthy reefs for settlement (Strader, Davies, & Matz, 2015; Tebben et al., 2015; Vermeij, Marhaver, Huijbers, Nagelkerken, & Simpson, 2010). Major urban and industrial infrastructures may mask or distort acoustic cues through noise, disrupt and interfere with chemical signals through sediment and toxic pollutant influx, inhibit light availability through high turbidity, induce sediment smothering of settlement‐cue‐surfaces, that is, coralline algae, and mislead planulae larvae toward settling near developed coastal areas hosting high intensity artificial light stimuli (Bauman et al., 2015; Codarin, Wysocki, Ladich, & Picciulin, 2009; Davies, Coleman, Griffith, & Jenkins, 2015; Leduc, Kelly, & Brown, 2004; Munday et al., 2009; Perez III, Rodgers, Jokiel, Lager, & Lager, 2014; Slabbekoorn et al., 2010; Tebben et al., 2015).

Excessive sedimentation in coral reef ecosystems as a result of anthropogenic influences is also an important parameter which affects coral larval recruitment and is one of the most widespread contemporary human‐induced perturbations on coral reefs (Bak & Engel, 1979; Bauman et al., 2015; Birkeland, 1977; Birrell, McCook, & Willis, 2005; Rogers, 1984). Heavy sedimentation in reef environments generally results in larger coral colonies through limited recruitment rates, strong algal competition, the inability of coral larvae to establish in shifting sediment regimes, and larval settlement on vertical surfaces (Birkeland, 1977; Rogers, 1990; Rogers et al., 1984). Sediment‐covered settlement surfaces may delay or prevent the settlement of coral larvae through the repression of vital cues (Graham, Baird, & Connolly, 2008), with the most commonly identified and effective sources of larval settlement and metamorphosis inducers being crustose coralline algae and reefal biofilms (Tebben et al., 2015). This is through the reciprocation of coral larvae to chemical extracts of crustose coralline algae, indicating that settlement substrata choice is critical for juvenile coral recruit survival (Sneed, Sharp, Ritchie, & Paul, 2014).

However, a study conducted at a site in Yanuyanu‐i‐Sau at the Great Astrolabe Reef south of Viti‐Levu, Fiji, revealed that high sedimentation rates in that area did not result in lower coral recruitment rates compared to other sites (Quinn & Kojis, 2008). Typically, it is acknowledged that sedimentation significantly reduces the potential reproductive capacity of corals (Kojis & Quinn, 1984); however, in this particular study, site similar coral recruitment rates were observed in comparison with another remote site throughout significantly higher sedimentation rates. The authors attributed the elevated recruitment rate to the presence of stable coral cover and rich species diversity, the provision of large amounts of larvae from adjacent fecund reefs, and the high number of spawning acroporid corals.

The concentration and type of sediment particles in suspension strongly influence water clarity and limit light availability for zooxanthellae photosynthesis; with water clarity itself directly determined by the extent at which waves re‐suspend sediments in shallow shelf seas, and increased phytoplankton activity as a result of sewage influx. Coastal reefs are known to flourish at relatively high levels of turbidity (DeVantier, De'Ath, G., Turak, E., Done, T., & Fabricius, K., 2006); however, they tend to be restricted to the upper 4–10 m because reduced zooxanthella photosynthesis rates reduce the coral growth at greater depths (Fabricius, 2011). Lower light levels at depths below 10 meters also inhibit the development of coral larvae by reducing the amount of energy made available to maturing embryos and ova. This was evident in a study which uncovered lower numbers of *Porites* larvae from colonies growing on reef ecosystems polluted by nutrient input and particulate matter in suspension, in comparison with the number of larvae sampled from a less polluted reef (Tomascik & Sander, 1987). Junjie, Browne, Erftemeijer, and Todd (2014) also found that due to the mechanical effects of sediment deposition, coral photosynthesis to respiration ratio (P/R ratio) and photosynthetic yield declined by as much as 21% and 18%–34% for corals exposed to sediments, compared to corals in a shaded control environment which saw declines of only 14% and 3%–17%. In a simulation study carried out under laboratory controlled conditions, Humphrey, Weber, Lott, and Fabricius (2008) reported that coral fertilization rates also decreased by more than 50% in correlation with increasing suspended sediment rates of 100 mg/L and decreasing salinity levels of 30 g/L. Moreover, there was a prevalence of development stage abnormalities in 100% of embryos and no fertilization at a salinity concentration of 28 g/L. Gilmour (1999) also reported that in *Acropora digitifera* a reef‐building coral species, suspended sediments of 50–100 mg/L substantially reduced larvae survivability and settlement; along with significantly reduced fertilization rates.

There is a general assumption that contemporary reefs located in nearshore light‐limited environments are typically in poor health, with the availability of information on the structure, community composition, and diversity of these shallow‐water reefs remaining scarce due to the challenging field conditions present in these locales (Morgan, Perry, Smithers, Johnson, & Daniell, 2016). Recently, however, the persistence of reefs in areas characterized by low light availability and periodic sediment smothering has been recognized, noting that coral reefs which grow and form in these settings can exhibit high live coral cover and species diversity (Gulliver, Palmer, Perry, & Smithers, 2013; Sanders & Baron‐Szabo, 2005). Furthermore, some reef corals have also evolved and adapted to high sediment‐laden reef environments (Potts and Jacobs, 2000), and occur in various regions of the world (Anthony & Larcombe, 2000).

Furthermore, it has been reported that the impact of these sediment induced conditions are not always negative, and that coral response to this stressor is species‐specific (Sofonia & Anthony, 2008). The usual rates of sediment deposition on coral reefs are reportedly around 10 mg cm^−2^ day^−1^, between 10 and 50 mg cm^−2^ day^−1^ for reefs under moderate to severe sedimentation, and >50 mg cm^−2^ day^−1^ for reefs experiencing extreme or catastrophic levels (Birkeland, 1997; Rogers, 1990). For coastal reefs which are typically located in shallow depths, the sedimentary structure can be volumetrically dominated by silts and muds (Smithers & Larcombe, 2003), with the reef ecosystem largely susceptible to wind‐driven sediment resuspension during storm events (Gulliver et al., 2013).

Despite nearshore contemporary reefs also being subject to nutrient stress as a result of inputs of poorly or inadequately treated sewage effluents into coastal waters, they are still capable of hosting high coral settlement, and diverse coral cover during these ongoing natural and anthropogenic perturbations (Lapointe et al., 2010). One of these mitigating mechanisms is through Chromophoric Dissolved Organic Matter (CDOM) through sedimentation, sewage, and river effluent influx into near shore reefs. Although CDOM has adverse localized impacts on nearshore reef ecosystems (Dupouy et al., 2014), it has been reported to lower coral mortality as a result of bleaching through the absorption of ultra violet radiation considerably more strongly than particulates such as detritus, phytoplankton, and visible radiation (Jokiel & Brown, 2004).

**Figure 1:**
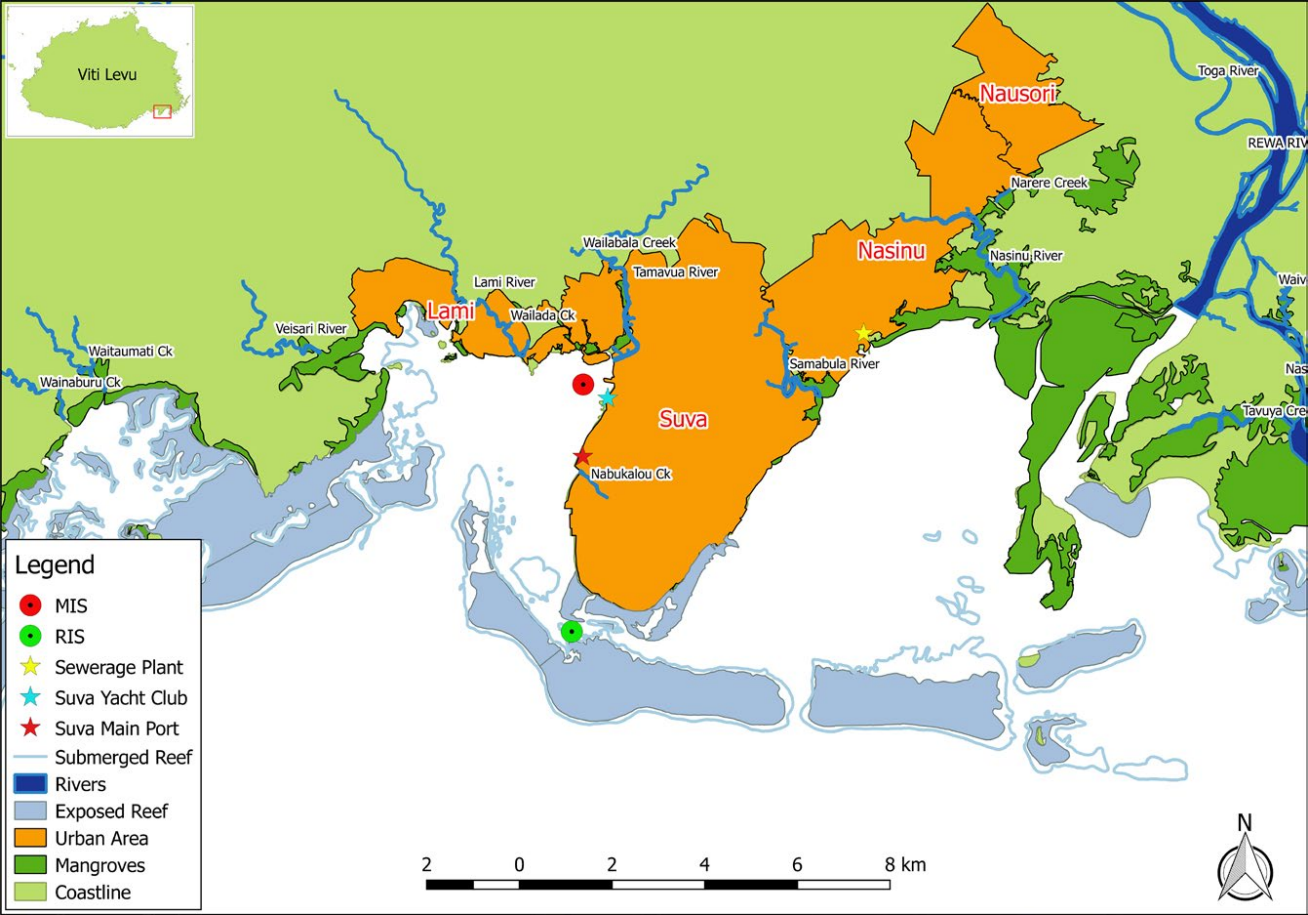
Site map showing study areas in the island of Viti Levu, Republic of the Fiji Islands. The red circular symbol denotes the MIS, with the immediately adjacent Suva Shipping Port depicted by the red star symbol. The close proximity of the Tamavua River mouth is visible, with the Suva Yacht Club represented by the aqua star symbol. The location of the nearby Nabukalou Creek which holds a documented history of pollution is also clearly visible

The port city of Suva is the capital of Fiji and is considered the largest urban center in the South Pacific, outside of Australia and New Zealand. Laucala Bay harbours the main large shipping port and most of the human settlements and industry of the region (Figure 1). Former larval recruitment studies in Suva Harbour were conducted offshore on the Suva Reef, and within the deeper Suva lagoon (Quinn & Kojis, 2008; Vave, 2013), with no emphasis placed on particulate organic matter content and associated light penetration, or in‐depth monthly monitoring of coral cover amidst sedimentation. For example, Quinn and Kojis (2008) found that the lowest recruitment rate was observed at a sediment influenced site within Suva Harbour in the deeper lagoon; however, to date, no previous study has evaluated coral recruitment in close proximity to the industrial area despite the existence of coral assemblages including reef‐building species from the genera *Acropora, Porites, Fungia* and *Pocillopora*.

It is vital to understand the extent to which anthropogenic inputs may interact with natural variation in coral recruitment and health, with these adverse stressors likely to have a significant effect on the successful settlement, establishment, and propagation of reef‐building corals. This study therefore aimed to examine the impacts of key anthropogenic pressure variables on coral health and recruitment using a comparison between a representative inshore locality and a naturally turbid reef (Figure 1). To achieve this our objectives centered upon determining coral spat recruitment rates, coral cover variation, and species diversity present between each reef, and correlating our findings with major environmental parameters in order to explore possible interactions and causal effects.

## 2 MATERIALS AND METHODS

### 2.1 Study sites

The Fiji Islands experience a distinct dry season between May to October, with the wet season typically occurring between November and April. Rainfall which ranges from 1,676 to 3,573 mm annually is highly seasonal, variable, and orographic and largely influenced by ENSO cycles and tropical cyclones in the summer months (Kumar, Stephens, & Weir, 2014; Lough, Meehl, & Salinger, 2011). Predominant southerly swells throughout the year, and easterly winds arising from March to December generate dominant semidiurnal tides with tidal ranges of 2.3 m (Australian Government Bureau of Meteorology, 2016). Surface currents are determined through ocean bathymetry, seabed geomorphology, presence of islands, as well as the shifting position of the South Pacific Convergence Zone (SPCZ).

The study was conducted at two inshore localities within Suva Harbour. The municipal site, hereafter designated MIS, consists of an inshore relic reef located ~375 m from the Suva Wharf, Walu Bay industrial area, yachts berthed at the Royal Suva Yacht Club, and several nearby floating ship repair dry docks. The site receives strong anthropogenic input in the form of consistent sedimentation input from the nearby Tamavua River, freshwater influxes through city sea wall storm water drains, dredging pressure, and reported chemicals from agricultural runoffs, and sewage effluents from the adjacent Nabukalou Creek (Naidu & Morrison, 1994; Prakash & Jokhan, 2013; Tabudravu, Gangaiya, Sotheeswaran, & South, 2002). Notably, midway through this study (6th December 2014), a major sewage spill discharged about 200 L/s of untreated waste water into Laucala Bay and this continued unabated for 18 days leading to a Government Environmental Emergency Declaration prohibiting swimming and fishing in the affected waters. The remote site (Dennis’ Patch), hereafter designated RIS, is characterized as a lagoon patch reef in the Suva region located at 18°10′S Latitude and 178°25′E Longitude. It holds a maximum depth of five meters with its reef zonation designated as a slope (Morris & Sykes, 2009).

### 2.2 Quadrat monitoring

#### 2.2.1 Coral cover

At each study site, five permanent quadrats were established, with permanent quadrat 1 established in the shallowest depth, and successive permanent quadrats being established in incrementally deeper depths down the reef slope. A 2 × 2 m portable quadrat was constructed from PVC pipes which were 20 mm in diameter, with sections of PVC pipe drilled at 0.5 m intervals in order to facilitate the forming of 16 sub‐quadrats with lengths of nylon rope and numbered underwater tags. Individual permanent quadrat locations were selected by placement of the portable quadrat atop of selected coral colonies and by driving four 3/8″ rebar iron markers into the substrate at each corner of the portable quadrat. The top left hand marker of each permanent quadrat was affixed with an aluminum tag which was punched with holes specifying the particular quadrat number.

A camera tripod stand was also fabricated according to English, Wilkinson, and Baker (1997) using welded ¼’’ iron rods along with an aluminum plate platform for camera placement. The tripod stand height ensured a distance of 60 mm from the substrate, which deviated from the 80 mm specification mentioned in English et al. (1997) in order to account for increased turbidity in the MIS. Laminated A4 sheet photographs displaying the correct orientation of the corners of each quadrat within quadrat markers were also used as an underwater guide to ensure correct placement of the portable quadrat within the quadrat markers. An Olympus Tough TG2 underwater digital camera with an attached FCON‐T01 Fisheye Converter lens was placed atop of the camera platform of the tripod stand, with the tripod stand then placed exactly atop of each sub‐quadrat and photographed in a left‐right direction until all 16 sub‐quadrats were photographed. Sampling could not be undertaken in both study sites for the period spanning December 2014–January 2015 due to a national environmental disaster which had arisen due to a major sewage spill disaster in the Suva Harbour area on December 6, 2014. Monitoring was resumed at the earliest allowable opportunity, which was in February 2015.

### 2.3 Image processing

Coral Point Count with Excel extensions (CPCe v.4.1) software was used to analyze coral cover statistics in relation to other major categories, for example, macro algae, sand etc., as well as coral species abundance and diversity. Each sub‐quadrat image was calibrated to scale (50 cm × 50 cm) and then overlaid with 20 software‐generated random points; with the life form features underlying each point subsequently identified. Processed sub‐quadrat photographs were then collectively analyzed for coral cover, and coral species diversity and abundance for each 2 × 2 m permanent quadrat. Data recovered every six months was analyzed and used to compare differences in coral cover and species diversity and abundance; in accordance with standard ecological monitoring practices (Hill & Wilkinson, 2004).

### 2.4 Coral recruitment

**Figure 2:**
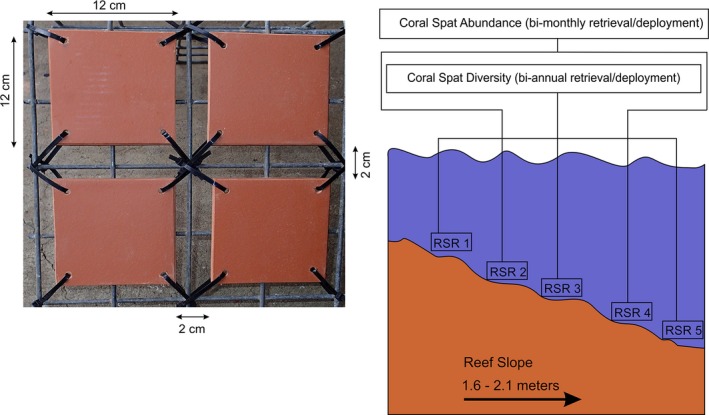
The left section shows terracotta tile placement and allowable spacing between tiles for each recruitment station rack in accordance with English et al. (1997). The right section illustrates the recruitment station rack deployment layout in both sites, in a linear formation and along an incrementally increasing depth profile toward the reef slope. For representative sampling across the study site, in each site RSR 2 and RSR 4 were allocated to examine coral spat abundance, with RSR 1, RSR 3, and RSR 5 designated for examining coral spat diversity

Terracotta tiles were unglazed and had corrugations present on the underside. Each study site had five recruitment station racks permanently secured to the ocean floor with U‐shaped hooks, with each rack deployed alongside each permanent quadrat (Figure 2). Tile collections were scheduled in line with predicted mass coral spawning dates for Fiji in 2014, which were determined in accordance with Mildner (1991), and found to occur between October 14th and November 13th. Settlement tiles were established one month before the commencement of spawning, with tile retrieval conducted at least within one month after spawning.

### 2.5 Settlement tile retrieval and recovery process

Settlement tiles were retrieved using wooden trays in order to minimize damage to settled spat during transport to the laboratory**.** Retrieved tiles were washed in running freshwater to remove sediments and algae; the cleaned tiles were completely immersed in labeled stainless steel trays filled with a 10% diluted bleach solution (CLOROX™, 8.25% Sodium Hypochlorite) and left to stand for 24 hr in order to remove organic material and reveal diagnostic skeletal features. Tiles were then washed with freshwater and allowed to dry at room temperature before being searched twice for coral spat in accordance with the protocol developed by Harriott (1992). Photographs of entire settlement tiles from each study category were taken for comparative visual estimates of calcareous algae coverage between sites.

### 2.6 Coral spat settlement and family level identification

An OLYMPUS™ SZ51 binocular microscope was used to detect coral spat on recovered tiles; each visible surface being searched at the lowest magnification setting (0.8×) in a “zigzag” fashion as demonstrated by Vave (2013), with the aid of a miniature fluorescent desktop lamp as the primary source of illumination. Corrugations present on the underside of the tile were intensively searched, and where applicable a small steel spatula was used to remove calcareous or organic matter in order to expose the underlying tile surface in an attempt to locate coral spat. Upon discovery of coral spat, the magnification was then increased to 4X and the spat identified to Family level based on skeletal structural features using the taxonomical method of Babcock, Baird, Piromvaragorn, Thomson, and Willis (2003), (see Supporting information Figure S1). Spat which could not be distinctly identified due to insufficient skeletal growth or due to damage to the corallite skeleton were grouped into an “Unidentifiable” category.

The shape of each identical square tile was taken as a right rectangular prism of sides equal to 12 cm and height 1 cm, with a fixed volume of 144 cm^3^. The surface area of each tile was therefore calculated using the formula: **A = 2(*wl*** **+ hl + *hw*)** giving a total surface area for individual tiles of 336 cm^2^. Total surface area relating to each spat count observation in each study site was calculated in order to make a correlation with total coral spat density for a fixed area, and during a given period of time.

### 2.7 Sediment analysis

**Figure 3:**
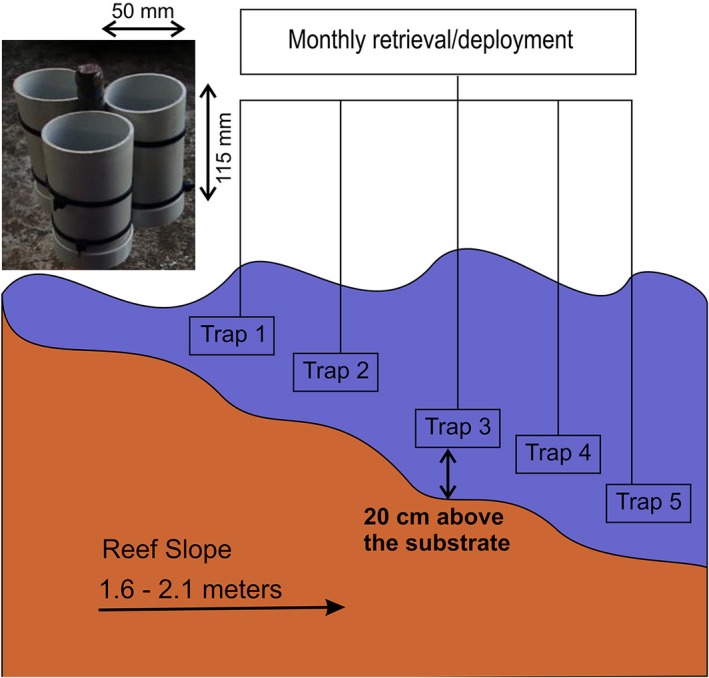
Sediment trap design and dimensions in accordance with English et al. (1997) depicted in the left panel, and field deployment layout and substrate placement shown in the right panel. One sediment trap which comprised of three cylindrical PVC collection tubes accompanied each permanent quadrat and recruitment station rack, in each site

Sediment traps were constructed according to guidelines recommended in English et al. (1997). One sediment trap was deployed alongside each of the five permanent quadrats in each of the two study sites (Figure 3). No baffles were placed at the mouth of PVC traps used in this study as it was deemed that their use may result in decreased trapping efficiency (Knauer & Asper, 1989).

Following retrieval, the entire contents of each PVC trap were transferred directly into clean 50 ml Eppendorf centrifuge tubes using a clean metal spatula. Distilled water was used to aid in the removal of the sediment sample from the PVC trap, and also to fill each tube up to the 50 ml mark. Algal growth and encrusting matter found along the inside of each trap was not collected for analysis; however, distilled water was used to thoroughly flush and retrieve any ensnared sediment matter from these growths in order to ensure unbiased determination of particulate organic matter (POM). Each sample was centrifuged for 10 min at 3,961 *g* and 26°C in a refrigerated centrifuge (Thermo IEC Centra CL3R). The supernatant was then poured off and the centrifuge tube filled to the 30 ml mark with distilled water. The tube was then shaken to stir the contents, and re‐centrifuged at 3,961 *g* for a further 10 min. This was done in order to ensure the removal of magnesium and calcium carbonate salts from the remaining sediment pellet.

Following centrifugation, the supernatant was discarded, and the sediment pellet transferred using a spatula and distilled water to a pre‐weighed ceramic crucible pre‐conditioned in a muffle furnace (Ceramic Engineering OELMEC) for 4 hr at 450°C. The crucibles were then allowed to cool in a desiccator for 30–40 min. Once cooled, the crucibles were placed collectively in a tray, and placed in a drying oven (OSK 9519A) for 12–14 hr at 60°C, or until a constant weight was achieved for individual crucibles containing the sediment pellets. The crucibles were then removed from the drying oven and placed immediately in a desiccator to cool for a further 30–40 min. The individual crucibles with pellets were then weighed and the net weight (in g/cm^2^) of the pellets recorded.

Following net pellet weight determination, the crucibles with sediment pellets were again placed into the muffle furnace for 4 hr at 450°C for ashing. The heated crucibles were then allowed to cool for 360 min within the furnace and subsequently transferred to a desiccator to allow for further cooling to ambient temperature. The crucibles were then re‐weighed and the net ashed weight in grams recorded after correction for crucible weight. Ash‐free dry weight (AFDW) was calculated using the difference between the dry‐weight and the ashed weight, which is equivalent to the (POM) and expressed as a percentage of the total dry weight.

The average daily sediment trap collection rate (ADCTR) in grams per day was calculated for each PVC pipe trap by dividing the total dry‐weight mass of sediments in each trap by the cross‐sectional area (cm^2^) of the trap mouth, and further dividing this figure by the number of days the particular trap was deployed in the study site (DeMartini et al., 2013; Storlazzi, Field, & Bothner, 2011). The cross‐sectional area of each sediment trap mouth was calculated using the formula: *A* = π*r* ^2^ and for a trap mouth diameter of 5 cm, the cross‐sectional area was 19.625 cm^2^.

### 2.8 Environmental parameters

Six Temperature and Light Data Loggers (HOBO^®^ Pendant^®^, model UA‐002‐64) were used to monitor these parameters at ten‐minute intervals twenty‐four‐hours daily, and for monthly logging periods throughout the 12‐month study. Data loggers were placed in pairs at each site in equidistant positions. In order to acquire an accurate representation of temperature and light intensity variation with depth at each study site, one logger in each study site was deployed in the shallow depth (Depth 1), with the other logger deployed in the deep depth (Depth 2).

Salinity and Dissolved Oxygen (DO), was measured at each site monthly using a YSI‐85 multi‐parameter probe. The equipment was first calibrated in terms of the correct height at sea level, with three measurements for each parameter then undertaken at varying depths: 0.3, 1.5, and 2.5 m. Mean values were then derived from these successive measurements.

### 2.9 Statistical analysis

All categories of data in this study, other than that of light intensity, Photosynthetic Photon Flux Density (PPFD), and temperature did not fulfil the conditions of normality and homoscedasticity, and were therefore analyzed using non‐parametric statistical tests in SPSS (v.21). For the coral spat family abundance and diversity categories, a differences analysis using the Mann–Whitney U test was conducted. In order to explore statistically significant associations between coral spat family abundances and diversity found in study sites and seasonality, the Pearson's chi‐squared test was employed. To investigate for the presence of significant differences between study sites for sediment dry‐weight, particulate organic matter, and average daily sediment trap collection rates, the Mann–Whitney *U* test was again performed. A parametric one‐way ANOVA differences test was used to investigate for the presence of any significant differences in light intensity between different depth profiles between and within study sites, with the Tukey HSD (Honestly Significant Difference) post hoc test being further employed in order to explore multiple comparisons between the different depth profile groups between and within study sites.

## 3 RESULTS

### 3.1 Environmental parameters

A significant difference in light intensity (*F*_3, 1442_ = 138.883, *p* < 0.01; one‐way ANOVA differences test) existed between similar depth profiles between study sites (Table 1). A highly significant interaction existed between the shallow depth profile in the MIS and the shallow depth profile in the RIS (total mean difference of 167.5 lum/ft^2^); and also between the deep depth profile in the MIS and the deep depth profile in the RIS (total mean difference of 171.4 lum/ft^2^) ;*p* < 0.01; Tukey HSD multiple comparisons post hoc test). An Independent Samples *t* test found a significant difference between the shallow and deep depth profile for the MIS (*F*_732, 682_ = 11.452, *p* < 0.01), but not for the RIS (*F*_732, 729_ *= *0.836, *p* > 0.01).

**Table 1:** One‐way ANOVA test results; total mean light intensity and daily mean PPFD values recorded annually for study sites at similar depth profiles; Depth 1: Shallow, Depth 2: Deep

Site and depth profiles	Total light intensity mean (lum/ft^2^)	Daily PPFD mean (mol m^−^^2^ day^−^^1^)	Significance value (interaction between groups)
MIS			0.000
Depth 1	5,821.34 ± 5.69	0.64 ± 0.05	
Depth 2	3,941.04 ± 4.53	0.48 ± 0.03	
RIS			
Depth 1	18,065.37 ± 10.19	2.16 ± 0.09	
Depth 2	17,196.56 ± 10.75	1.96 ± 0.09	

Similarly, a significant difference in PPFD calculated from light intensity data between similar depth profiles existed between study sites (*F*_3,144_ = 128.804, *p* < 0.01; one‐way ANOVA differences test; Table 1). Further tests revealed a highly significant interaction between the shallow depth profile in the MIS and the shallow depth profile in the RIS (total mean difference of 1.52 mol m^−2^ day^−1^), and also between the deep depth profile in the MIS and the deep depth profile in the RIS (total mean difference of 1.48 mol  m^−2^ day^−1^; *p* < 0.01; Tukey HSD multiple comparisons post hoc test). An Independent Samples *t* test found a significant difference between the shallow and deep depth profile for the MIS (*F*_732, 648_ = 11.805, *p* < 0.01), but not for the RIS (*F*_733, 732_ = 0.526, *p* > 0.01).

**Figure 4:**
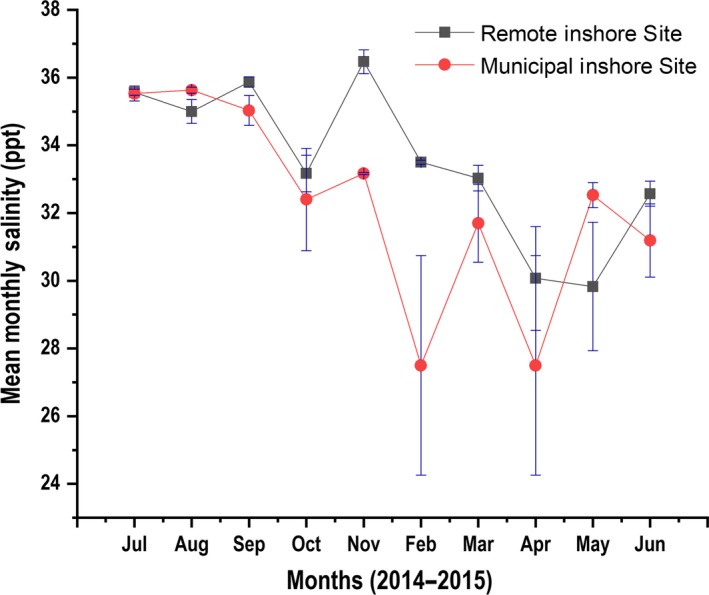
Mean monthly salinity values depicted by the red trend line for the Municipal Inshore Site, and the blue trend line for the Remote Inshore Site

**Figure 5:**
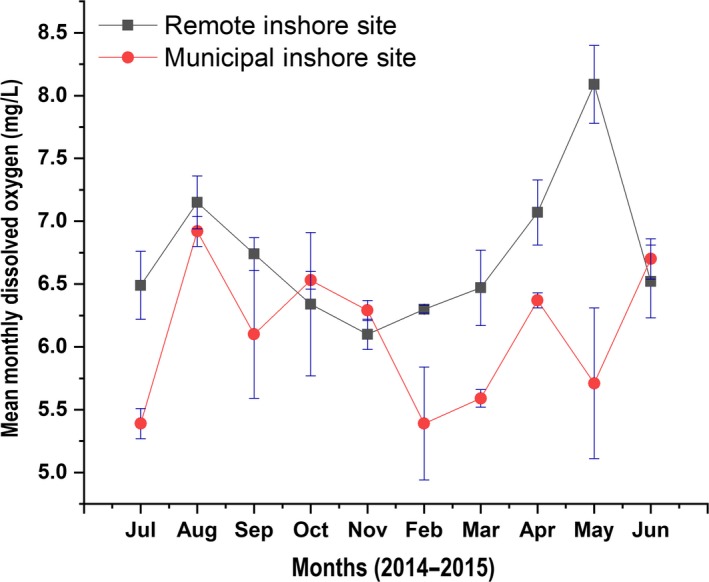
Mean monthly dissolved oxygen values depicted by the red trend line for the Municipal Inshore Site, and the blue trend line for the Remote Inshore Site

**Figure 6:**
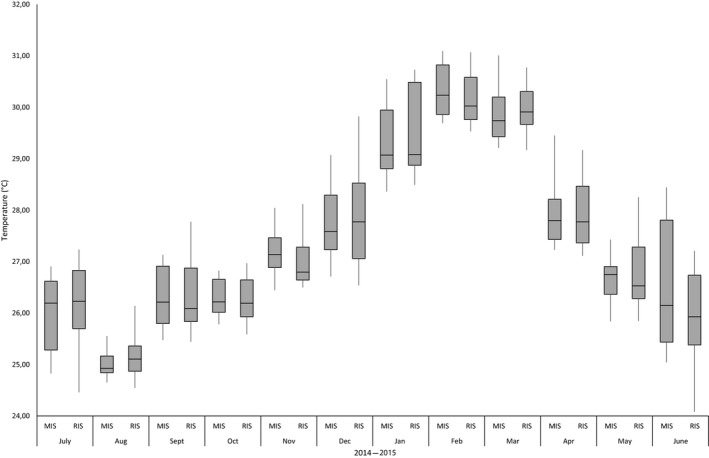
Mean water temperature denoted as a monthly comparison between the Municipal Inshore Site and the Remote Inshore Site

Monthly mean salinity values were significantly different between sites (*p* < 0.05) only for November 2014 and February 2015 (Figure 4). Monthly mean dissolved oxygen values between sites were also significantly different (*p* < 0.05) for July 2014, March 2015, and May 2015 (Figure 5). No significant differences were found in monthly mean water temperatures between sites (*p* > 0.05, one‐way ANOVA test), except for September 2014 (Figure 6).

### 3.2 Monthly sedimentation rates

**Figure 7:**
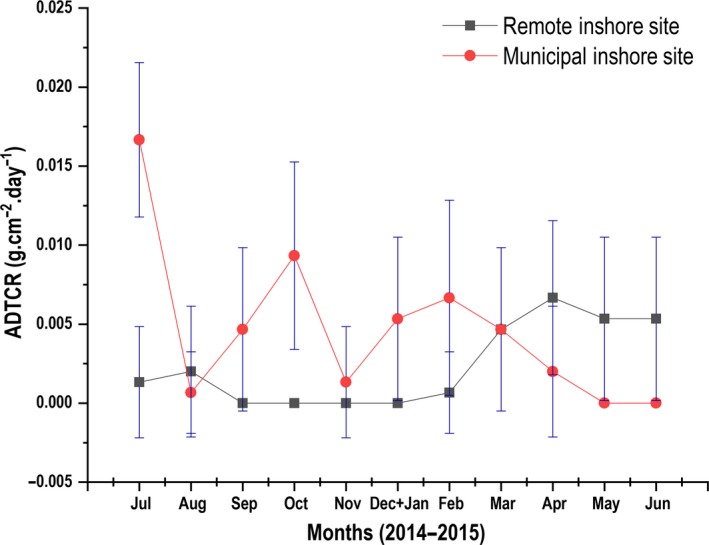
Mean Average Daily Trap Collection Rate (ADTCR) calculated from monthly sediment dry‐weight values for each site, and indicated for the Municipal Inshore Site through the red trend line, and for the Remote Inshore Site through the blue trend line

**Figure 8:**
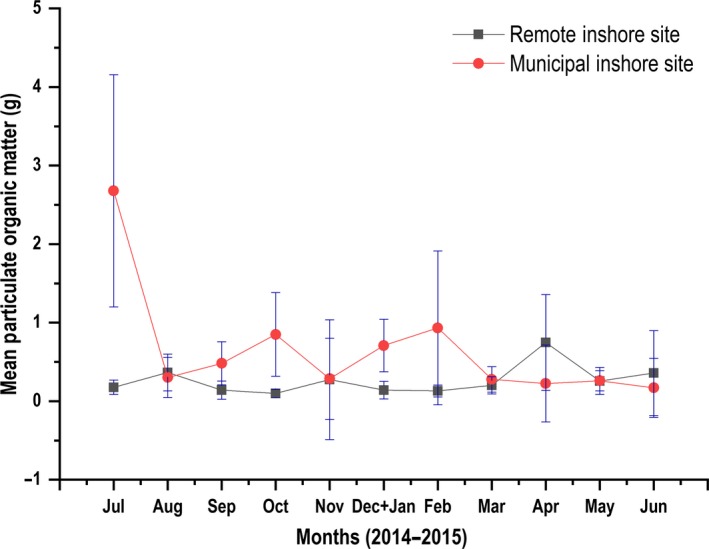
Mean Particulate Organic Matter (POM) indicated for the Municipal Inshore Site through the red trend line and for the Remote Inshore Site through the blue trend line. Particulate Organic Matter values respective to each site were derived from sediment dry‐weight and ash‐free dry weight (AFDW)

The MIS recorded the highest total monthly accumulated sediment weight (657.14 g cm^2^) after a monitoring duration of twelve months. Sediments retrieved from this site were found to be silt dominated. In the same period, the RIS recorded significantly less sediment dry‐weight (371.52 g cm^2^) (*U* = 10,733.5, *p* < 0.05; Mann–Whitney U test). A two‐way repeated measures ANOVA conducted after (log base 10 transformation) (including Within Subjects‐Effects and Within Subjects‐Contrasts) revealed a significant difference in terms of sediment dry‐weight between sites (*F*_1,1_ = 595.167, *p* < 0.01). Further tests also revealed significant interactions (*p* < 0.01) between the factors (site and time). ADCTR in terms of sediment dry‐weight were significantly higher (*U* = 11,296.5, *p* < 0.01; Mann–Whitney U test) for the entire duration of monitoring in the MIS compared to the RIS (Figure 7). A statistically significant difference in the percentage of POM composition in sediment dry‐weight was found between the MIS and the RIS for the duration of the study (*U = *8,329.0, *p* < 0.01; Mann–Whitney U test) (Figure 8).

### 3.3 Coral cover

For the MIS, a statistically significant difference in mean ranks existed between the pair of observations for month 1 and 7 in terms of coral cover percentage (*p* = 0.043; Wilcoxon's multiple comparisons post hoc test). No statistically significant difference in mean ranks were found between month 1 and 12 (*p* = 0.500), and month 7 and 12 (*p* = 0.686). The RIS recorded a statistically significant difference in coral cover percentage between month 7 and 12 (*p* = 0.043) and month 1 and 12 (*p* = 0.043), with no significant difference found between months 1 and 7 (*p* = 0.144) (see Supporting information Figure S2). Noteworthy differences in macro algae percentages were recorded between sites in all monitoring months; Month 1: MIS‐5.56%, RIS‐0%; Month 7: MIS‐25.68%, RIS‐0.06%; Month 12: MIS‐27.81%, RIS‐0%.

A total of 15 coral species were identified in the MIS, compared to 14 species for the RIS in month 1, 11 species in the MIS and 13 species in the RIS in month 7, and 15 species in the MIS and 13 species in the RIS in month 12 (Table 2; Supporting information Figure S3). In terms of species diversity between sites comparatively higher Shannon–Weaver Diversity Indices were recorded in the MIS for all monitoring months in relation to the RIS; Month 1: MIS‐1.34, RIS‐0.69; Month 7: MIS‐1.30, RIS‐0.68; Month 12: MIS‐1.49, RIS‐0.72.

**Table 2:** Coral species percentages of monthly coral cover

Coral species	Site and monthly abundance (mean % of Coral Cover)					
	Month 1		Month 7		Month 12	
	MIS	RIS	MIS	RIS	MIS	RIS
*Acropora elseyi*	1.86	0.38	1.09	0.32	0.82	0.31
*Acropora hyacinthus*	0	0.25	0	0.25	0	0.25
*Acropora micropthalma*	1.51	2.90	2.88	2.59	1.52	2.64
*Acropora millepora*	3.73	0	0	0	0.64	0
*Acropora nobilis*	6.12	0.76	0.76	0.57	0.71	0
*Acropora pulchra*	0.20	0	0.63	0	0	0
*Favites* sp.	0.07	0	0	0	0.13	0
*Favites halicora*	0	0.25	0	0	0.13	0
*Fungia repanda*	1.77	0.19	2.66	0.38	2.21	0.44
*Leptastrea purpurea*	0	0.90	0.76	0	0.96	0.06
*Millepora tenella*	0	13.42	0	12.13	0	10.53
*Porites australiensis*	0	0	0	0.06	0	0.44
*Porites cylindrica*	0	2.83	0	2.21	0	2.08
*Pocillopora damicornis*	2.05	0.56	1.91	0.19	2.67	0.38
*Porites lobata*	5.39	15.03	6.40	14.39	15.14	5.71
*Pocillopora*	0.07	0	0	0	0	0
*Porites* sp.	0.52	0	0	0	0	0
*Porites rus*	2.19	31.57	1.72	29.57	0.82	23.46
*Platygyra sinensis*	0	0.06	1.72	0.06	0.06	0.25
*Psammocora* sp.	0.07	0	0	0.06	0.13	0.13
*Pavona varians*	0.13	0	0	0	0.25	0
*Stylophora pistillata*	0.39	0	1.39	0	1.70	0
*Turbinaria reniformis*	0	0.13	0	0	0	0

### 3.4 Coral spat recruitment

A total of 242 coral spat were discovered and positively identified from both study sites from July 2014 to July 2015, on a combined number of 144 terracotta settlement tiles. A subtotal of 110 spat were found from the coral spat “Abundance” category, compared to 132 spat recorded from the “Diversity” category. Interestingly, the MIS recorded a total coral spat density for the entire duration of the study which was not significantly different (*p* = 0.317), from the total coral, spat density found in the remote inshore site; MIS: 106 coral spat on a surface area of 6,720 cm^2^ from 20 tiles, compared to 132 coral spat from the RIS on the same surface area and number of tiles. Four coral families were photographed and positively identified throughout the course of the study, namely: *Acroporidae*,* Pocilloporidae*,* Poritidae*, and *Lobophyllidae* with developmental stages from between 2 and 6 months for some families recorded and verified in accordance with Babcock et al. (2003). Coral spat retrieved from the MIS also demonstrated a cryptic settlement preference between corrugations present on the underside of settlement tiles.

In the bimonthly interval abundance sampling regime, the MIS recorded 49 annual coral spat, compared to the RIS with 78 annual coral spat recorded. Coral spat from the Family *Lobophyllidae* were notably absent from this sampling category, as this taxon was exclusively found in the six‐monthly interval sampling regime. A significant difference in the abundance of Family *Poritidae* coral spat was found between the two study sites (*U* = 605.0, *p* = 0.022; Mann–Whitney *U* test). A higher abundance of this family was found in the RIS, and for all other coral families no significant difference in coral spat recruitment between sites was detected (*p* > 0.05; Mann–Whitney U test) (Table 3).

**Table 3:** Total coral spat numbers from six bimonthly “Abundance” collection intervals; MIS: Municipal Inshore Site; RIS: Remote Inshore Site

Coral spat Family	Coral spat sum in sites		Mann–Whitney mean ranks	
	MIS	RIS	MIS	RIS
*Acroporidae*	3	3	40.50	40.50
*Pocilloporidae*	31	28	39.61	41.39
*Poritidae*	33	12	35.63	45.38
*Lobophyllidae*	0	0	38.85	42.15
*Unidentifiable*	11	6	40.50	40.50

In the coral spat biannual diversity sampling regime, 132 coral spat were found and identified from both study sites in the same period. A total of 87 spat were recorded at the MIS while the RIS recorded 83 coral spat. Coral spat from the Family *Lobophyllidae* were exclusively found after six months of undisturbed artificial substrate availability. No statistically significant differences between study sites in the abundance of coral spat from any Family were found (*p* > 0.05; Mann–Whitney U test; Table 4).

**Table 4:** Total coral spat numbers from two biannual “Diversity” collection intervals; MIS: Municipal Inshore Site, RIS: Remote Inshore Site

Coral spat Family	Coral spat sum in sites		Mann–Whitney Mean Ranks	
	MIS	RIS	MIS	RIS
*Acroporidae*	9	28	21.88	27.13
*Pocilloporidae*	31	9	25.27	23.73
*Poritidae*	20	11	26.50	22.50
*Lobophyllidae*	3	5	23.94	25.06
*Unidentifiable*	24	14	26.96	22.04

## 4 DISCUSSION

Our results indicate that environmental stressors such as sedimentation and sewage pollution do not necessarily result in a reduction in coral cover, or a change in the recruitment rate of coral spat. Most field studies investigating the effects of sewage pollution on coral reef ecosystems have been short‐term and limited in scope (Pastorok & Bilyard, 1985), and report that sewage‐stressed ecosystems typically experience a decrease in coral cover, taxonomic richness, water clarity, larval recruitment, and decreased calcification rate as a result of the sewage effluent (Laws & Redalje, 1982). Conversely, our year‐long recruitment study discovered that 106 coral spat successfully settled on artificial substrate (67.2 m^−2^) in an environmentally stressed near‐shore site, and in the midst of numerous anthropogenic stressor influences which are intrinsic to urban and industrial areas. Previous recruitment studies undertaken in Suva Harbour have focused only on larval diversity through biannual settlement tile collections, and revealed comparatively lower recruitment farther offshore in Suva Channel and at a larger depth (Quinn & Kojis, 2008). Where larval abundance has been examined, monitoring duration is limited to six months and has been conducted offshore off the Suva Reef (Vave, 2013).

Corals are very vulnerable in their recruitment stage to stress as newly settled coral larvae and young colonies are extremely sensitive to adverse variables not conducive to their survival. In contrast to our results, very little coral larval settlement was observed taking place on sediment‐covered surfaces in laboratory and field experiments (Hodgson, 1990; Te, 1992, 2001 ), and also within the natural environment (DeMartini et al., 2013; Larcombe, Costen, & Woolfe, 2001). Perez III et al. (2014) also report through laboratory experiments that larval recruitment is completely abated in sediment film environments below 0.9 mg/cm^2^ (0.047 mm thick), and Quinn and Kojis (2008) revealed a total of 5 recruits per m^−2^ in a reportedly turbid site in Suva channel, Fiji. However, our results for Suva Harbour demonstrate that despite a total annual sediment yield of 657.14 g cm^2^ for the stressed MIS, 2 recruits/m^2^ were found to settle in this site throughout this period. The RIS which experiences a lesser degree of anthropogenic disturbance recovered 132 spat amidst a total annual sediment yield of 371.52 g cm^2^. In addition to this, excessive sediment films forming on settlement tiles in the MIS caused decreased calcareous algae colonization on the tile surfaces compared to tiles from the RIS (see Supporting information Figure S4). The former finding was similar to results reported in Bauman et al. (2015), where periods of peak settlement were observed despite year‐round recruitment, and through sustained anthropogenic disturbance (high sedimentation and turbidity). The peak in POM percentage in sediment dry‐weight observed at the MIS from the month of March 2015 (0.75 g) was likely attributed to increased phytoplankton concentrations in the water column arising as a consequence of increased nutrient availability due to the December 2014 sewage spill disaster in Suva Harbour. The sole significant difference in the recruitment found in the abundance sampling regime for the *Poritidae* coral spat between the two study sites may imply a preference of habitat for this family as settling larvae in a highly disturbed equatorial reef were found to comprise only 6% of the total abundance, in comparison with other spat Families (Bauman et al., 2015).

We speculate that the influx of coral larvae is one of the most important factors influencing the persistence of the relic reef ecosystems found inshore. This could be possible through the seeding of coral larvae from nearby coral reef ecosystems, or through self‐seeding which can be a primary factor in the resilience of isolated reef systems (Gilmour, Smith, & Brinkman, 2009). Our findings also correlate with recent work undertaken by Bauman et al. (2015), and demonstrate that settlement patterns observed in the MIS are working to support sediment‐tolerant local adult assemblages. In the biannual recruitment study, the absence of a statistically significant difference in the diversity of any particular coral spat family category between study sites highlights the resilience capacity of the MIS reef ecosystem in terms of a clear persistence in a sub‐optimal environment. A clear demonstration of coral post‐settlement survival is evident from our study. Family *Lobophyllidae* spat demonstrated structural features indicative of six months of settlement, that is, two or more septal cycles along with the presence of a rudimentary columella. Family Poritidae recruits exhibited large and typical adult corallite structures, along with Pocilloporidae recruits which displayed skeletal features typically seen at two months of development (Babcock et al., 2003).

Our observation that coral spat from the Family *Lobophyllidae* were exclusively found after six months of undisturbed artificial substrate availability substantiates the notion that there is a seasonal influence to the gametogenic cycle in this family. For example, Soto and Weil (2016) found that oogenesis for the species *Isophyllia sinuosa* and *Isophyllia rigida* correlated with warm local sea surface temperatures. Our study, reports the presence of spats belonging to the Family *Lobophyllidae* only in December 2014 which is after the major coral spawning period for Fiji and throughout warm temperate seasonality.

It has been reported that the amount of available light intensity and spectral quality affects the settlement density, attachment position, and survival of coral larvae from zone‐specific coral species in sub‐tropical coral communities (Ho & Dai, 2014; Maida, Coll, & Sammarco, 1994; Minton, Lundgren, & Pakenham, 2007; Mundy & Babcock, 1998). Despite this, our results for the MIS show an annual total of 49 coral spat recovered from six bimonthly intervals in the abundance study throughout relatively low mean daily light, compared to the RIS which recorded 78 annual coral spat for the same study with relatively higher mean daily light intensity (Table 3).

We conclude that the MIS received sufficient daily PAR to allow the survivability of selected coral species since maximum photosynthesis rate in the zooxanthellae algal cells of *Pocillopora damicornis* and *Porites lobata* takes place at a light intensity (PPFD) of approximately (200 µmol/m^−2^ s and about 350 µmol/m^−2^ s) respectively; and with other coral species also displaying photosynthetic efficiency in low light conditions (Kinzie III & Hunter, 1987; Riddle, 2013). It can also be assumed that light intensity is relatively restricted in the MIS due to sediment induced conditions and periodic incidences of sediment resuspension as a likely result of consistent boat traffic originating from the Suva Yacht Club and adjacent industrial area. It can also be inferred that light intensity in the RIS was also impaired as we found that the dominant coral species contributing to coral cover at the RIS was *Porites rus* which has a preference for turbid waters.

The reduction in salinity from 35–28 ppt is expected to significantly undermine successful coral fertilization and also results in a 50% impairment in the development of active and motile swimming planulae larvae (Humphrey et al., 2008). We surmise this as being a possible causal factor adversely affecting larval recruitment in the MIS, as mean monthly salinity levels for November 2014 and February 2015 were significantly lower compared to the RIS. Salinity values for both February 2015 and April 2015 were 27.50 ppt. respectively. These observed salinity levels may have been attributed to the heavy rainfall which occurred during the wet season period in the Fiji Islands for that year (see Supporting information Figure S5). Monthly mean DO was also observed to be significantly lower for the MIS for July 2014, March 2015, and May 2015 compared to the RIS, and although we did not discover DO variation within the range of 4 mg/L which would indicate severe macro‐algal dominance (Haas, Smith, Thompson, & Deheyn, 2014), variations in DO values observed in this study may be a contributing factor to the abundance of macro algae present in the MIS ecosystem.

The diverse coral cover and recruitment rate of viable coral spat seen in the MIS may be attributed to adaptation mechanisms, as some nearshore reef environments along the Great Barrier Reef coastline, Australia, were reported to display high coral cover indicating adequate physiological responses to periodic stress events (Anthony & Larcombe, 2000). Possibly maximum sedimentation is only experienced in short periods which allows for potential recovery periods between sub‐lethal sediment influxes, heterotrophy on suspended particles during reduced light levels and reduced rates of photosynthesis (Anthony & Fabricius, 2000; Anthony & Larcombe, 2000; Browne, 2012; Rogers, 1983). Furthermore, it may also reflect photo acclimation by near shore corals in order to increase photosynthetic efficiency during incidences of high sedimentation. Rapid regrowth of remnant corals or asexual reproduction could also possibly play an influential role in the maintenance of the local reef population in the MIS (Bauman et al., 2015). In addition to this, the cryptic settlement behavior of spat could also be contributing to post‐settlement survival, and sediment load mitigation (Wham, Ning, and Lajeunesse (2017).

The adaptation of corals to varying sedimentation loads and short‐term exposure, in addition to reduced water quality throughout continuous coastal development has been readily documented (Browne, 2012; Fisk & Harriott, 1989; Mwachireya, McClanahan, Hartwick, Cote, & Lesack, 2015; Rogers et al., 1984), along with that of adult coral tolerance to eutrophic conditions and the ability to compete with macro‐algal populations (Fabricius, 2011). Fleshy and fast‐growing macro algae are opportunistic organisms which benefit from disturbance and thrive in high nutrient and eutrophic environments (Anderson & Garrison, 1995). They readily outcompete living corals for space (Bellwood, Hughes, & Hoey, 2006; Birkeland, 1977), and gain competitive advantage by serving as carriers of coral diseases (Littler & Littler, 2013; Nugues, Smith, Hooidonk, Seabra, & Bak, 2004). Macro algae such as Sargassum spp. can coexist with corals in healthy reefs (Schaffelke, 1999); however, minimal nutrient increases may serve to exceed critical thresholds and stimulate macro‐algal biomass production and simultaneously inhibit corals (Littler & Littler, 1984).

Coral larvae may be attracted toward near‐shore areas hosting diverse reef organisms for settlement, and demonstrate positive responses to different cues including acoustic signals of a healthy reef consisting of fish calls and grunts, and that of snapping shrimps (Dixson, Abrego, & Hay, 2014; Lecchini et al., 2018; Vermeij et al., 2010). The Suva Harbour municipal site is directly exposed to ranging magnitudes of noise pollution from large ships and small boats from the adjacent major Suva Port, floating dry docks, the Suva Yacht Club, and drilling and construction noises from the nearby industrial area. This modified acoustic environment may mask important natural cues used by coral larvae as is the case in reef fish (Codarin et al., 2009; Slabbekoorn et al., 2010). More in‐depth studies are required here in order to further evaluate and understand the correlations involved in this anomaly.

Despite this natural affinity toward healthy reef environments for settlement, coral larvae also display an indifference toward sediment polluted near‐shore areas and are able to seek out suitable substrate by settling on high and low levels of sediment‐covered surfaces in response to physical and algal‐chemical cues (Tebben et al., 2015), and by using cilia to crawl along the sediment‐covered substrate; essentially removing sediment and forming tracks which are then used by other larvae for successful settlement (Perez III et al., 2014). Certain planulae larvae are also able to settle and survive in sediment affected environments where other species would perish by relocating to alternative areas after settlement through reversible metamorphosis (Richmond, 1985). For example, it has been documented that larvae of *Pocillopora damicornis* are capable of retracting all tissue from the coral skeleton, reverting back to the planktonic form and resettling a second time if they are stressed within three days of settlement (Richmond, 1985).

While coral larvae settlement is influenced by physical parameters associated with reef surfaces (topography, color, sound) and chemical cues (microbial biofilms, predator and prey odors, and algae); larval settlement and metamorphosis in response to these cues is selective in sub‐optimal habitats (Dixson et al., 2014; Tebben et al., 2015; Vermeij et al., 2010). It has been reported that in structurally complex, or low light environments, the detection of chemical cues and signals by aquatic organisms is impaired though the disruption of olfaction and chemosensory abilities (Leduc et al., 2004; Munday et al., 2009). This considerably affects homing, microhabitat selection, and the ability to perceive basic environmental stimuli (Leduc, Munday, Brown, & Ferrari, 2013). Light variation and light color is therefore an important cue used by coral larvae to discern depth and settlement surface orientation (Strader et al., 2015), and to identify optimum habitats for settlement and growth (Mundy & Babcock, 1998). For example, a recent study suggests that artificial light prevalent in urbanized and developed coastal environments can both encourage and inhibit the settlement of coral larvae on hard surfaces in these near‐shore areas (Davies et al., 2015).

## 5 CONCLUSIONS

To the best of our knowledge, the long‐term evaluation of seasonal abundance and diversity of coral larvae, coral cover, sedimentation, light intensity, and temperature in close proximity to a major urban center in the Pacific region has not been reported. This study discovered that consistent coral larval settlement rates were occurring in an area experiencing anthropogenic disturbances, a documented history of pollution (Naidu & Morrison, 1994), low light intensity levels, and high sedimentation and POM. Our results indicate that coral larvae may not distinguish between the significantly different light, sedimentation, and POM conditions found between the studied sites. Here, we also report annual variability in salinity, dissolved oxygen and temperature, and propose a possible attenuation with larval recruitment impairment in an inherently sub‐optimal setting. However, further evidence which could be attained from extensive future studies are required to justify the findings of this study. The inclusion of offshore study sites may also serve to provide a stronger statistical comparison between site results. The noteworthy anomalies in our findings should also be investigated through genetic analysis to test whether the observed physiological tolerance is a plastic response from standing genetic diversity present beyond this relic reef, or whether it is the consequence of local adaptation of a potentially strongly selected population. The adaptive responses of these corals to environmental stressors could then be analyzed through translocation experiments.

## CONFLICT OF INTEREST

The authors declare no competing financial interests.

## AUTHOR CONTRIBUTIONS

R.L., R.H.R., and A.D.R.N.Y. designed the study, R.L. and R.H.R. established the study sites, R.L. and C.R. performed the sample collections, R.L. carried out experiments and conducted analyses supervised by S.K. and C.R. R.L., C.R., S.K., and A.D.R.N.Y. wrote the manuscript; and all authors read and approved the manuscript.

## DATA ACCESSIBILITY

Data pertaining to this study has been deposited in Mendeley. *Ronal Lal* https://dx.doi.org/10.17632/j3jvdpyvj4.1
