# Supplementary material for: The effects of a stressed inshore urban reef on coral recruitment in Suva Harbour, Fiji
Source: Ecol Evol. 2018 Nov 20;8(23):11842–56. doi: 10.1002/ece3.4641 (PMC6303754; doi:10.1002/ece3.4641)
Supplement: Supplementary file 1 [file ECE3-8-11842-s001.docx]

**SUPPLEMENTARY INFORMATION**

“The effects of a stressed inshore urban reef on coral recruitment in Suva harbour, Fiji"

Ronal Lal, Stuart Kinninmonth, Antoine D. R. N’Yeurt, Ralph H. Riley& Ciro Rico

Contents:

[**S1: Coral spat photomicrographs: top-left: Poritidae; top-right: Lobophyllidae; bottom-left:** 2](#_Toc517780156)

[**S2: Coral cover in monitoring months** 3](#_Toc517780158)

[**S3: Coral species diversity in monitoring months** 4](#_Toc517780159)

[**S4: Settlement tile calcareous algae cover between sites.** 5](#_Toc517780160)

[**S5: Average monthly rainfall: Laucala Bay, Fiji (June 2014 - June 2015).** 7](#_Toc517780161)

# **S1: Coral spat photomicrographs: top-left: Poritidae; top-right: Lobophyllidae; bottom-left: Pocilloporidae; bottom-right: Acroporidae**


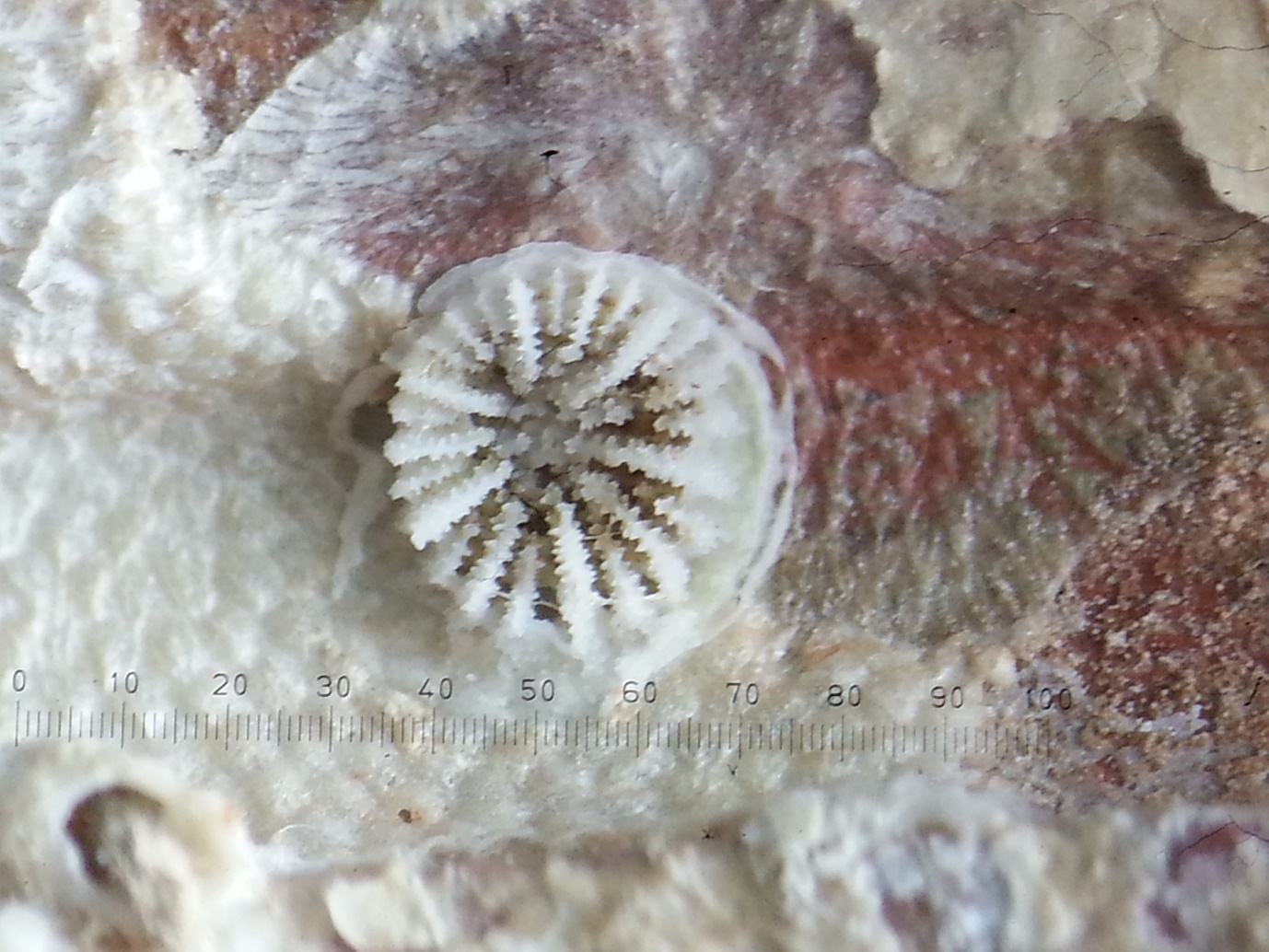

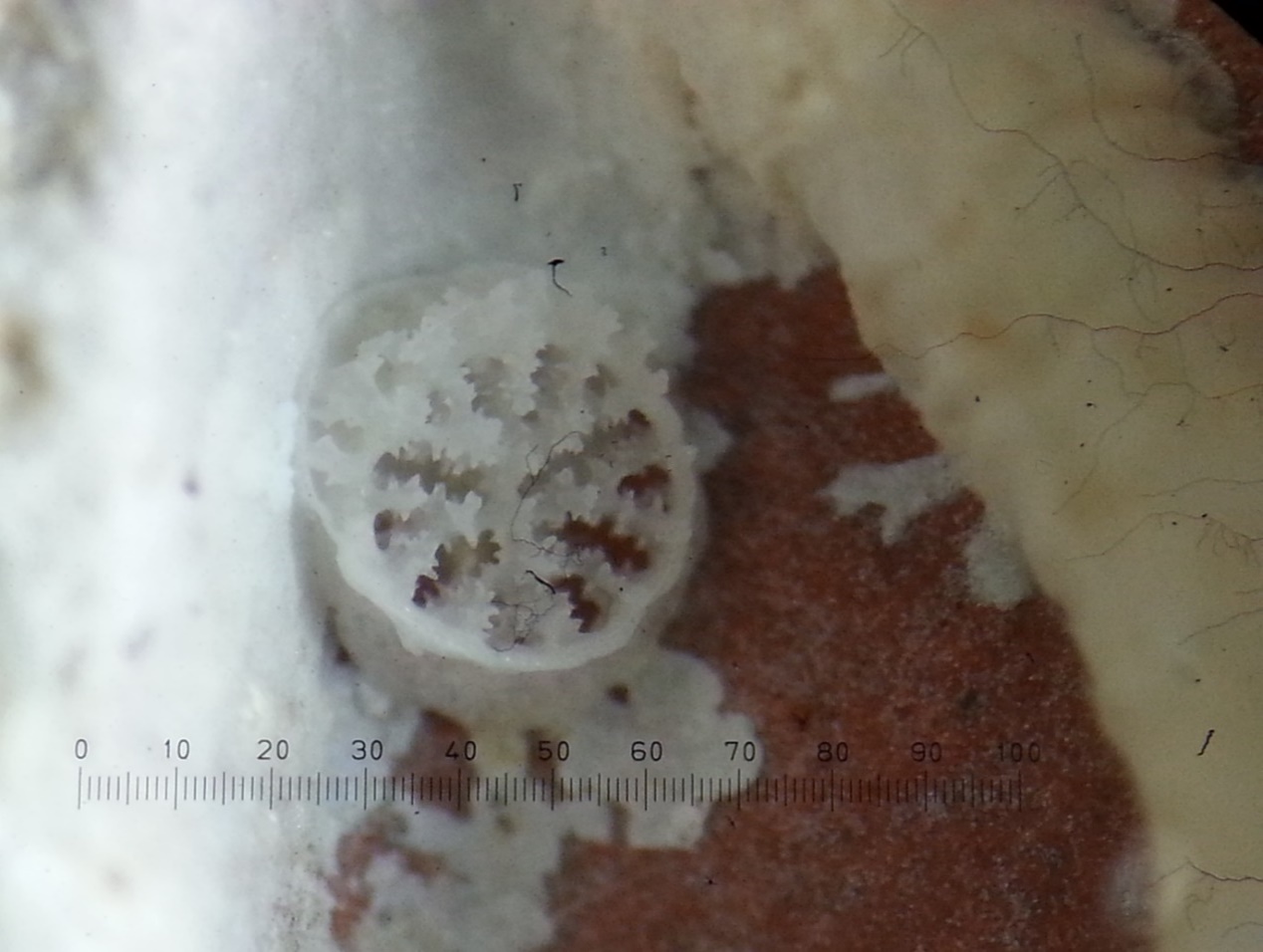

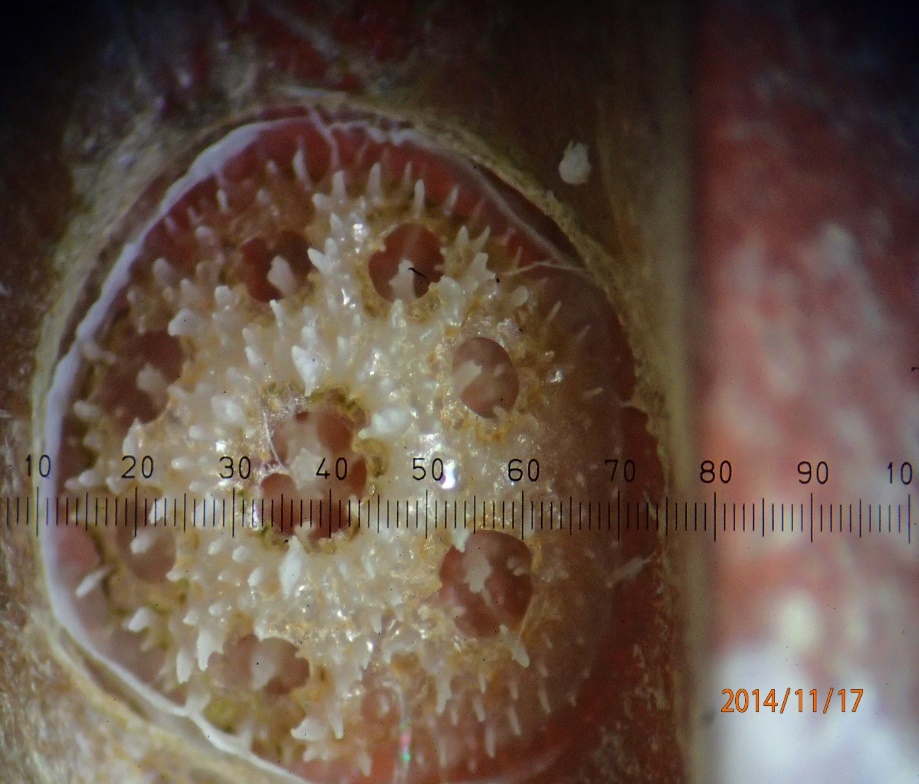

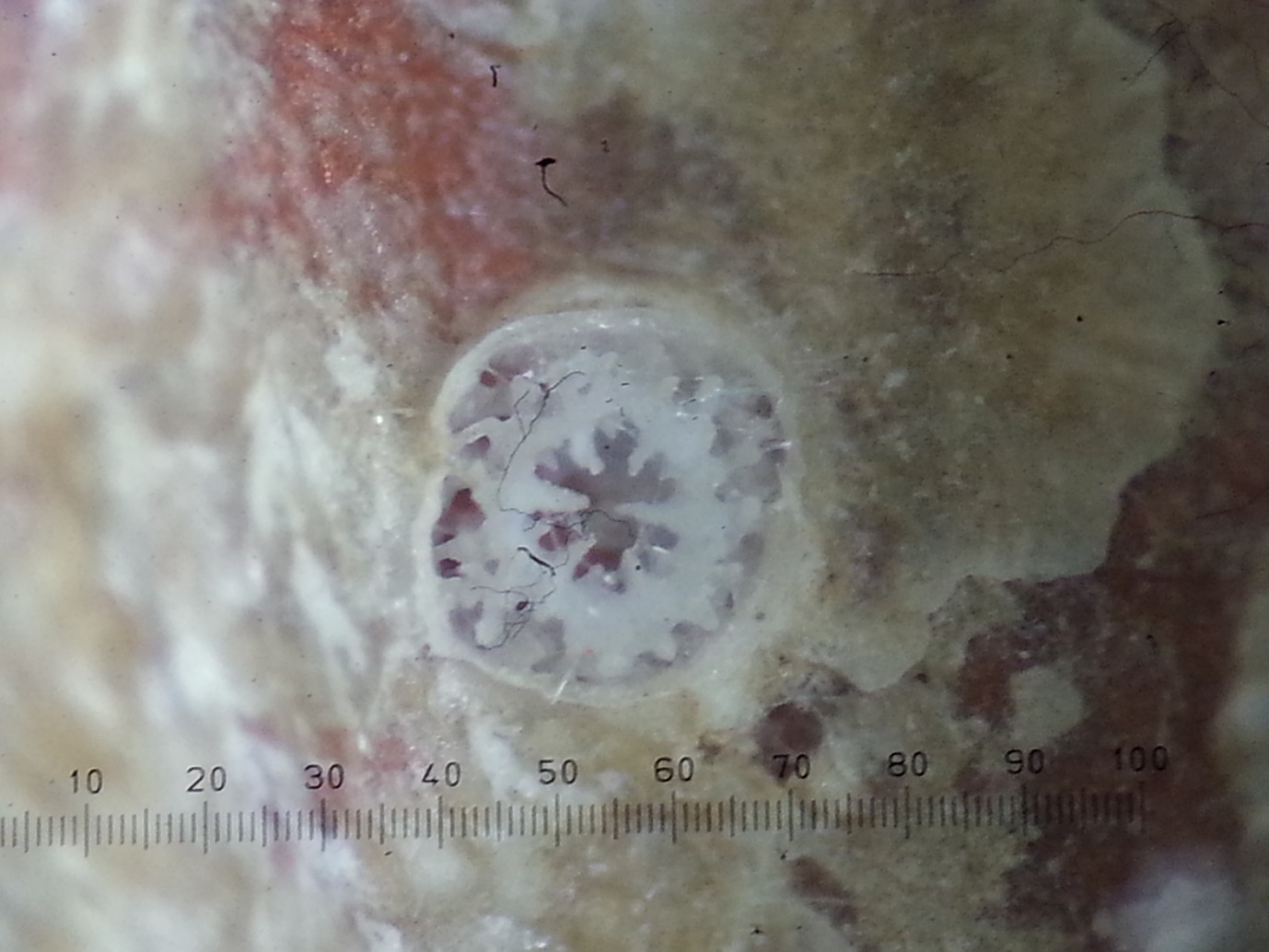


# **S2: Coral cover in monitoring months**


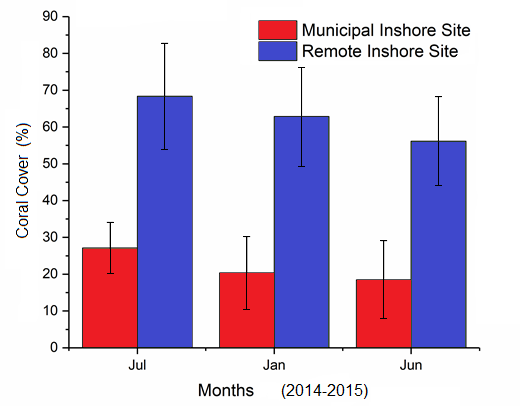


# **S3: Coral species diversity in monitoring months**

Coral species diversity was extrapolated from coral cover data acquired for each monitoring month: Month 1 (a); Month 7 (b); Month 12 (c).


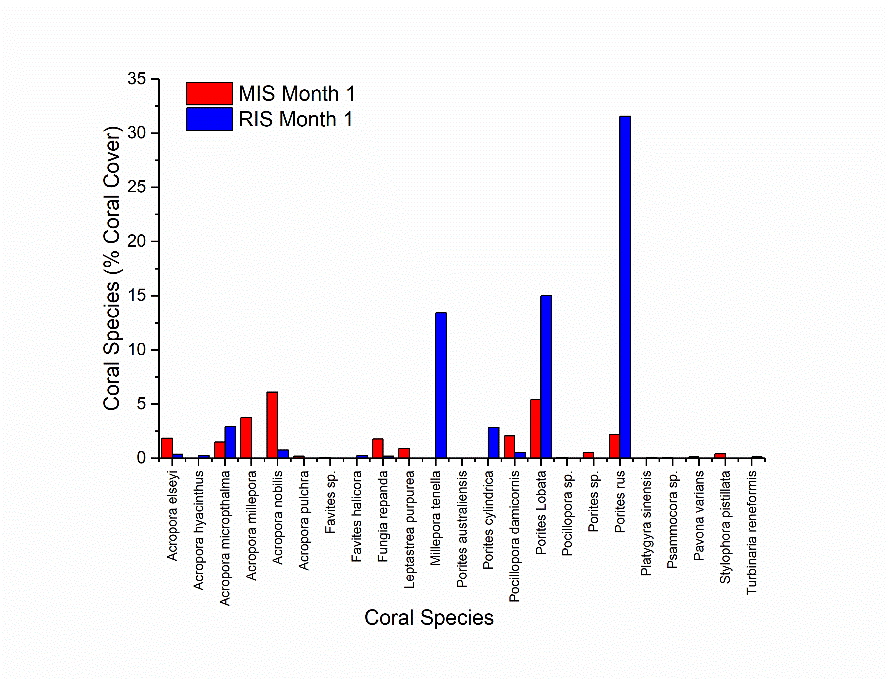

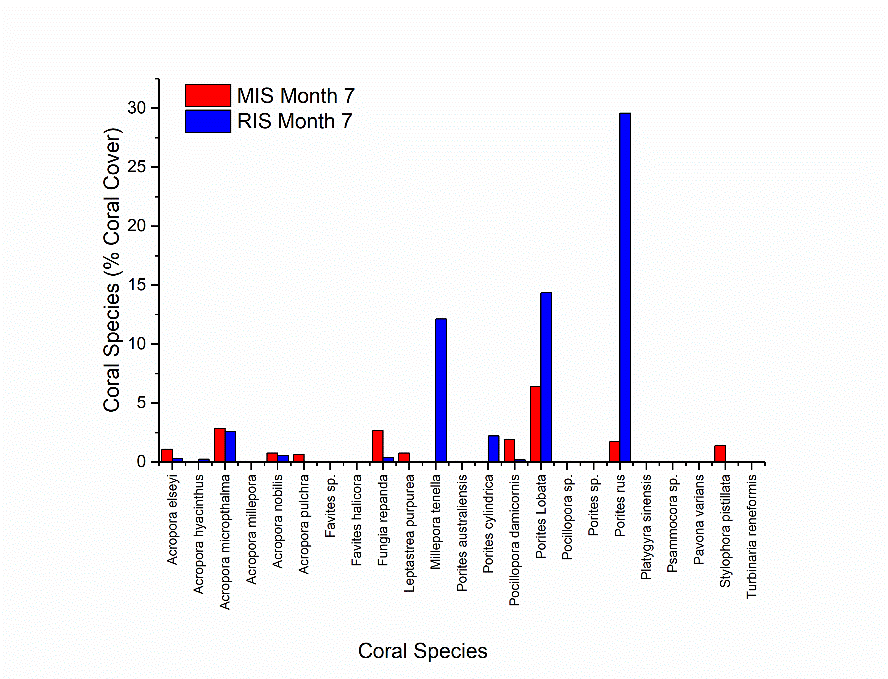

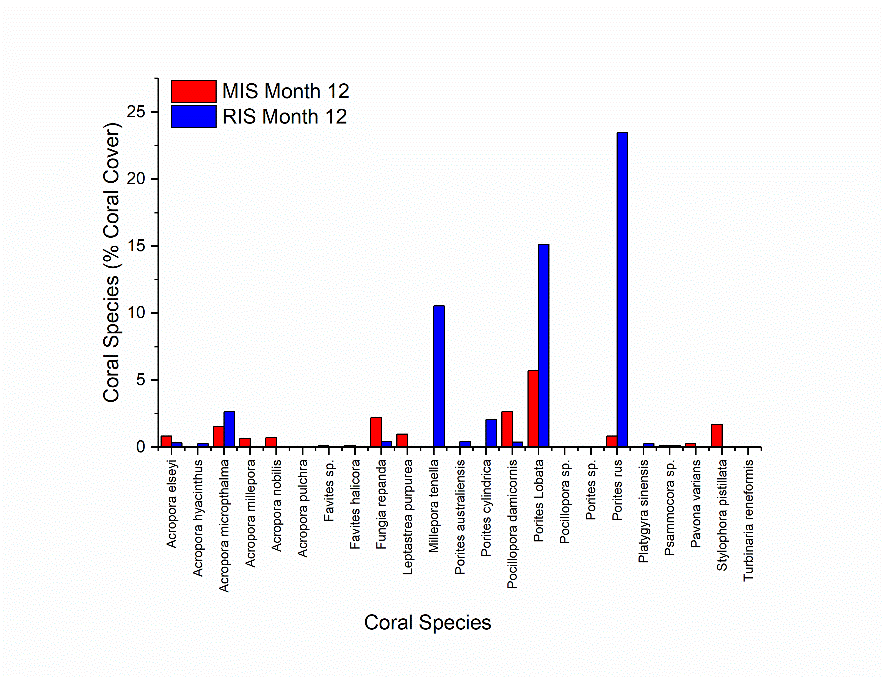


# **S4: Settlement tile calcareous algae cover between sites.**

Photo 1: Municipal Inshore Site, frontal view. Collected 24/11/14


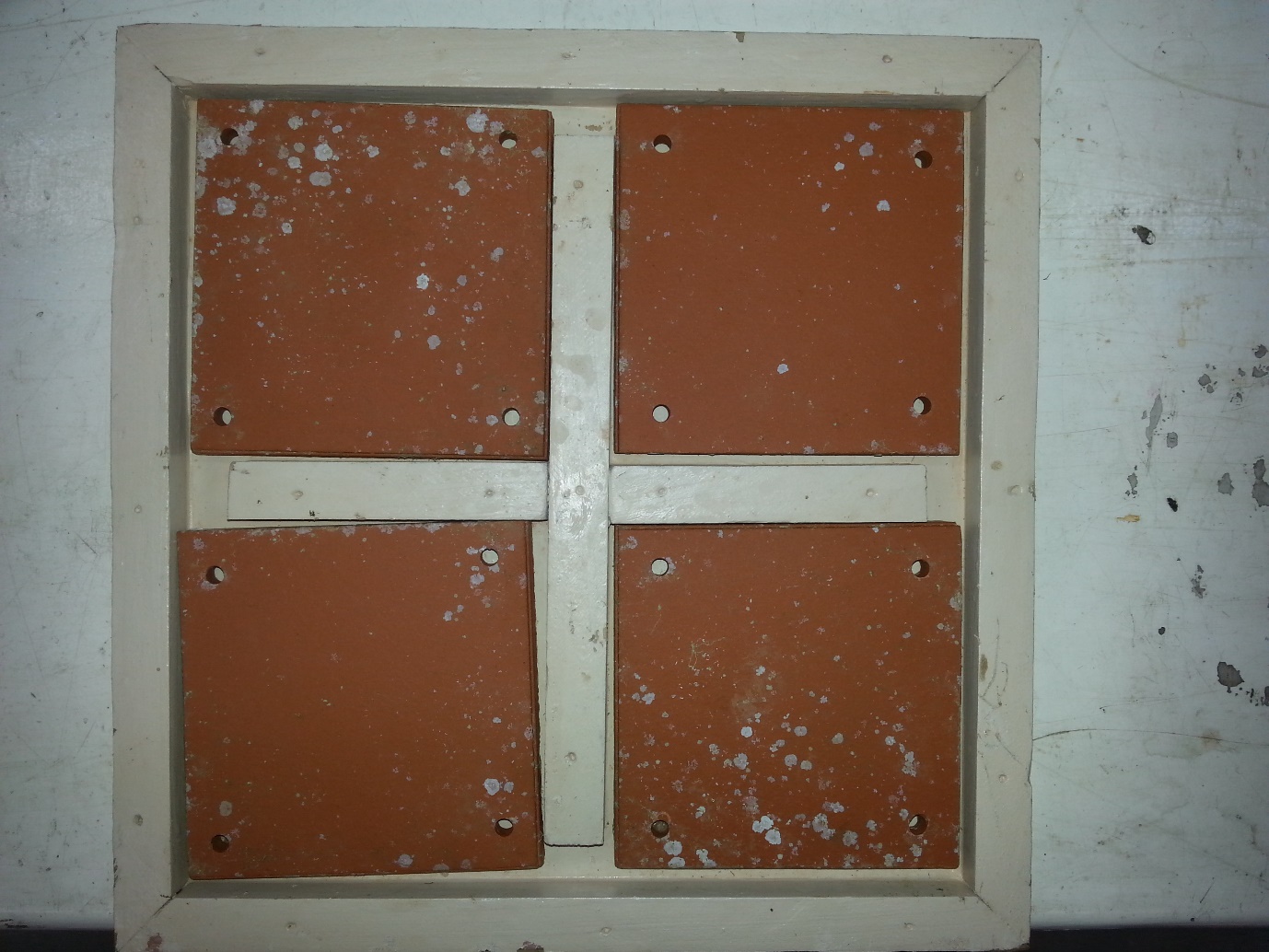


Photo 2: Remote Inshore Site, frontal view. Collected 13/11/14


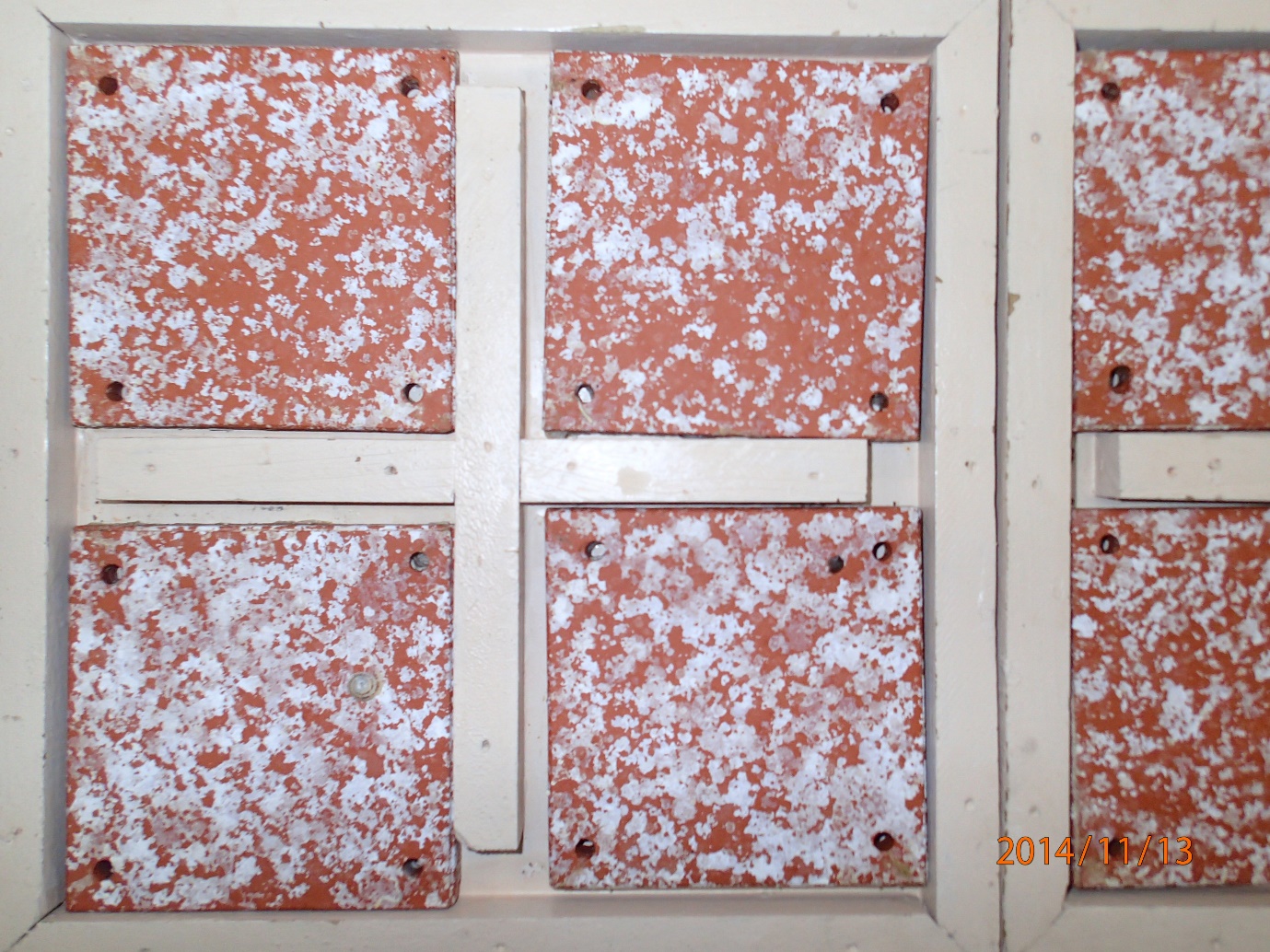


# **S5: Average monthly rainfall: Laucala Bay, Fiji (June 2014 - June 2015).**


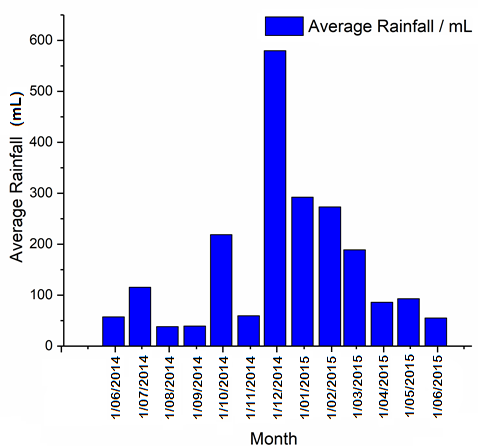


References

THE FIJI METEOROLOGICAL SERVICE OFFICE. Wind Speed and Rainfall, (Data Request Reference No. MET 49/2)
